# Radiographic Evaluation of Scoliosis in Pediatric, Adolescent, and Young Adult Patients: Associations Between Curve Severity, Localization, and Skeletal Maturity

**DOI:** 10.3390/diagnostics16203353

**Published:** 2026-10-15

**Authors:** Alpár Pászka, Zsuzsánna Pap

**Affiliations:** 1 Department of Nephrology, Mures County Clinical Hospital, 540072 Targu Mures, Romania; paszka_alpar@freemail.hu; 2 Department of Anatomy and Embryology, George Emil Palade University of Medicine, Pharmacy, Science, and Technology of Targu Mures, 540142 Targu Mures, Romania; 3 Radiology Department, Dora Medicals, 540327 Targu Mures, Romania

**Keywords:** scoliosis, Cobb angle, Risser stage, pediatric spine, radiology

## Abstract

**Background/Objectives**: The vertebral column is exposed to significant mechanical loads, exceeding 8000 N at certain levels due to bipedal posture and locomotion. These forces may contribute to structural alterations during growth. Scoliosis is among the most common structural spinal conditions in children and adolescents, with reported prevalence ranging from 0.5% to 5.2%. Our study aims to evaluate the radiographic characteristics of scoliosis in pediatric, adolescent and young adult patients and to analyze associations between curve severity and anatomical and developmental parameters. **Methods**: This retrospective cross-sectional study included 233 patients (152 females, 81 males) examined between August and October 2023. Cobb angle, curve localization, curve direction, Risser stage, and spina bifida occulta were assessed. Associations between variables were analyzed using chi-square and Fisher’s exact tests, and multivariable linear and ordinal regression models. **Results**: Scoliosis (Cobb angle ≥ 10°) was identified in 62.2% of patients within this referral cohort. A statistically significant association was observed between curve localization and scoliosis severity (*p* = 0.007), with thoracic curves being present more frequently with higher Cobb angles. No statistically significant associations were found between scoliosis severity and sex or curve direction. Spina bifida occulta was detected in 11.7% of scoliotic patients. Skeletal maturity followed expected age-related patterns. In multivariable ordinal logistic regression across the entire cohort, primary thoracic curve localization (aOR = 4.64, *p* < 0.001), female sex (aOR = 3.06, *p* < 0.001), and older age (aOR = 1.18, *p* = 0.002) were independently associated with higher scoliosis severity categories, whereas Risser stage and spina bifida occulta were not. In subgroup multivariable linear regression restricted to thoracic curves, higher Risser stage (β = −3.11°/stage, *p* = 0.041) and older age (β = 2.12°/year, *p* = 0.013) were independently associated with continuous Cobb angle magnitude. **Conclusions**: Scoliosis severity is associated with anatomical curve localization and skeletal maturity demonstrates age-appropriate ossification patterns. These findings support the value of integrated radiographic assessment, including Cobb angle evaluation and Risser staging, in the diagnostic work-up of pediatric, adolescent, and young adult scoliosis.

## 1. Introduction

The investigation of spinal disorders has been the subject of extensive studies over the past 70 years, largely because a significant proportion of the global population is affected by some form of spinal pathology. According to literature, scoliosis is the most common structural spinal deformity [1]. International data indicate that the prevalence of scoliosis ranges between 1.5% and 3%, while its occurrence during childhood and adolescence varies between 0.5% and 5.2% [2,3]. There is a lack of precise epidemiological data for Romania. However, according to literature, this condition is among the most prevalent structural spinal deformities in Europe [4].

In approximately 80–85% of cases, the etiology of scoliosis remains unknown, and the condition is most commonly detected during adolescence.

While adolescent idiopathic scoliosis is presumed to have a complex polygenic background associated with temporary neuromuscular imbalance during rapid growth [5,6,7,8,9], secondary forms (15–20%) may stem from underlying neuromuscular, genetic, or metabolic conditions [7,8,9,10]. Many long-term complications can be prevented if scoliosis is diagnosed at an early stage in pediatric and adolescent patients [6,11]. Clinical examination is indispensable for screening young individuals and identifying those who require further radiological assessment to confirm the diagnosis of scoliosis [8,12,13].

The evaluation of scoliosis is primarily performed using conventional radiographic imaging, which facilitates and accelerates diagnostic decision-making [11,12,13,14]. Following diagnosis and treatment initiation, follow-up imaging are essential to monitor therapeutic efficacy [8,11,12,15,16,17,18]. Although individual parameters such as Cobb angle and Risser staging are routinely assessed, the combined characterization of curve severity, anatomical curve localization, skeletal maturity, and posterior arch defects (spina bifida occulta) within the same referral cohort remain poorly captured in local populations.

Previous research demonstrates that curve severity and progression risk are strongly influenced by an interplay between skeletal maturity, anatomical curve patterns, and demographic profiles [6,19,20,21]. Recent literature highlights the necessity of multivariable analytic models to evaluate the independent associations of these factors while controlling potential confounders such as age and sex [22,23,24]. Specifically, multivariable risk stratification models have established that main thoracic curve localization and lower skeletal maturity stages (Risser 0–2) represent key independent correlates of higher curve magnitude [20,21,22,23,24,25,26]. However, most multivariable studies focus on strict prospective adolescent idiopathic scoliosis cohorts, whereas cross-sectional referral populations across a broader developmental age spectrum remain less characterized [22,27]. Therefore, this study aims to evaluate the integrated associations between curve severity, curve localization, spina bifida occulta levels, and skeletal maturity in a referral-based radiographic cohort.

We hypothesized that thoracic localization and lower skeletal maturity (Risser stages 0–2) are significantly associated with higher Cobb angles and severe scoliosis categories, even after adjusting to age and sex.

## 2. Materials and Methods

### 2.1. Study Population and Eligibility Criteria

**Figure A1:**
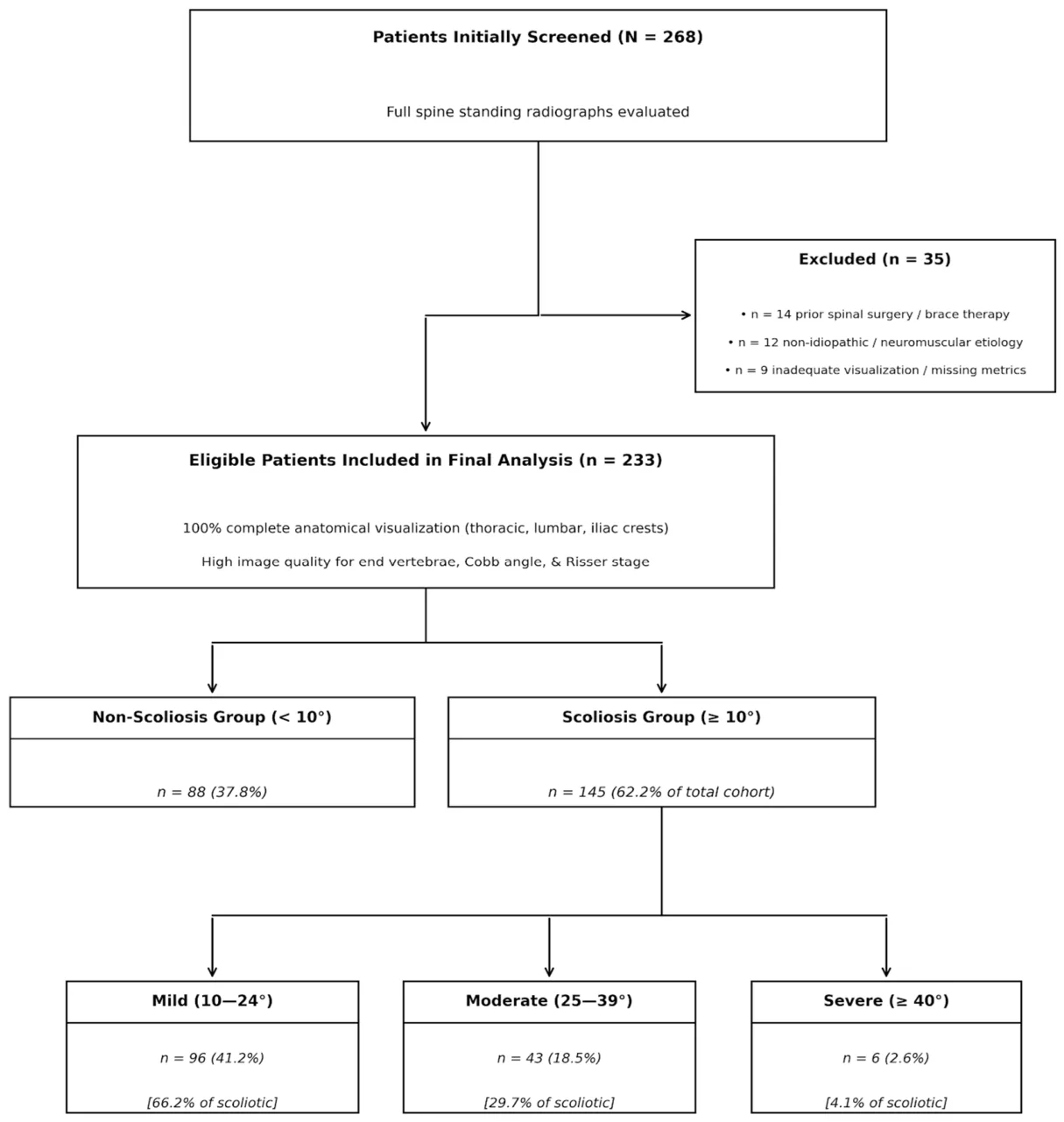
Participant flow diagram of screening, exclusion, and cohort stratification.

This retrospective observational single-center study analyzed standing anteroposterior (AP) spinal radiographs obtained between August and October 2023 at the Dora Medicals Radiology Clinic in Târgu Mureș, Romania. The study population comprised pediatric, and adolescent patients referred to radiological evaluation due to clinical suspicion of scoliosis, trunk asymmetry, or postural impairment during routine screening. To ensure a homogeneous cohort of idiopathic etiology, non-idiopathic forms—including congenital vertebral malformations, neuromuscular disorders, syndromic conditions, or prior spinal surgery—were systematically excluded during initial screening. A total of 233 consecutive patients were included in the final analysis, comprising 152 females and 81 males. Patients presenting with Cobb angles < 10° (n = 88) were designated as a non-scoliosis referral comparison group drawn from the same clinical referral pipeline, rather than healthy population control to allow comparative analysis against the scoliosis group (n = 145, Cobb angle ≥ 10°) (Figure A1) [28]. To account for sex-specific physiological differences in pubertal timing and skeletal maturation, age stratification was analyzed separately for female and male cohorts. Specifically, the pubertal growth spurt, peak height velocity, and iliac apophyseal ossification (Risser staging) occur approximately 1.5 to 2 years earlier in females than in males [29,30,31,32,33,34]. Applying uniform chronological age categories across sexes would misclassify developmental stages relative to skeletal maturity; therefore, sex-stratified age groupings were implemented to preserve physiological alignment with peak growth spurt windows and curve severity. Because the study population spans a broad developmental age range (5.8–25.4 years), findings in very young children and young adults are considered within their specific developmental contexts and are not treated as directly equivalent with homogeneous adolescent idiopathic scoliosis (AIS) screening cohorts.

Inclusion criteria were:

(1) availability of complete spinal radiographs including the thoracic spine, lumbar spine, and iliac crests.
(2) high image quality permitting precise determination of end vertebrae, Cobb angle, and Risser stage.
(3) patient age between childhood and young adulthood (0–25 years).
(4) radiographs obtained during the predefined study period.

Exclusion criteria were:

(1) incomplete, motion-degraded, or technically inadequate radiographs.
(2) prior spinal surgery or instrumentation.
(3) duplicate or follow-up examinations of the same patient during the study frame.

### 2.2. Radiological Assessment and Measurement Reliability

**Figure 1:**
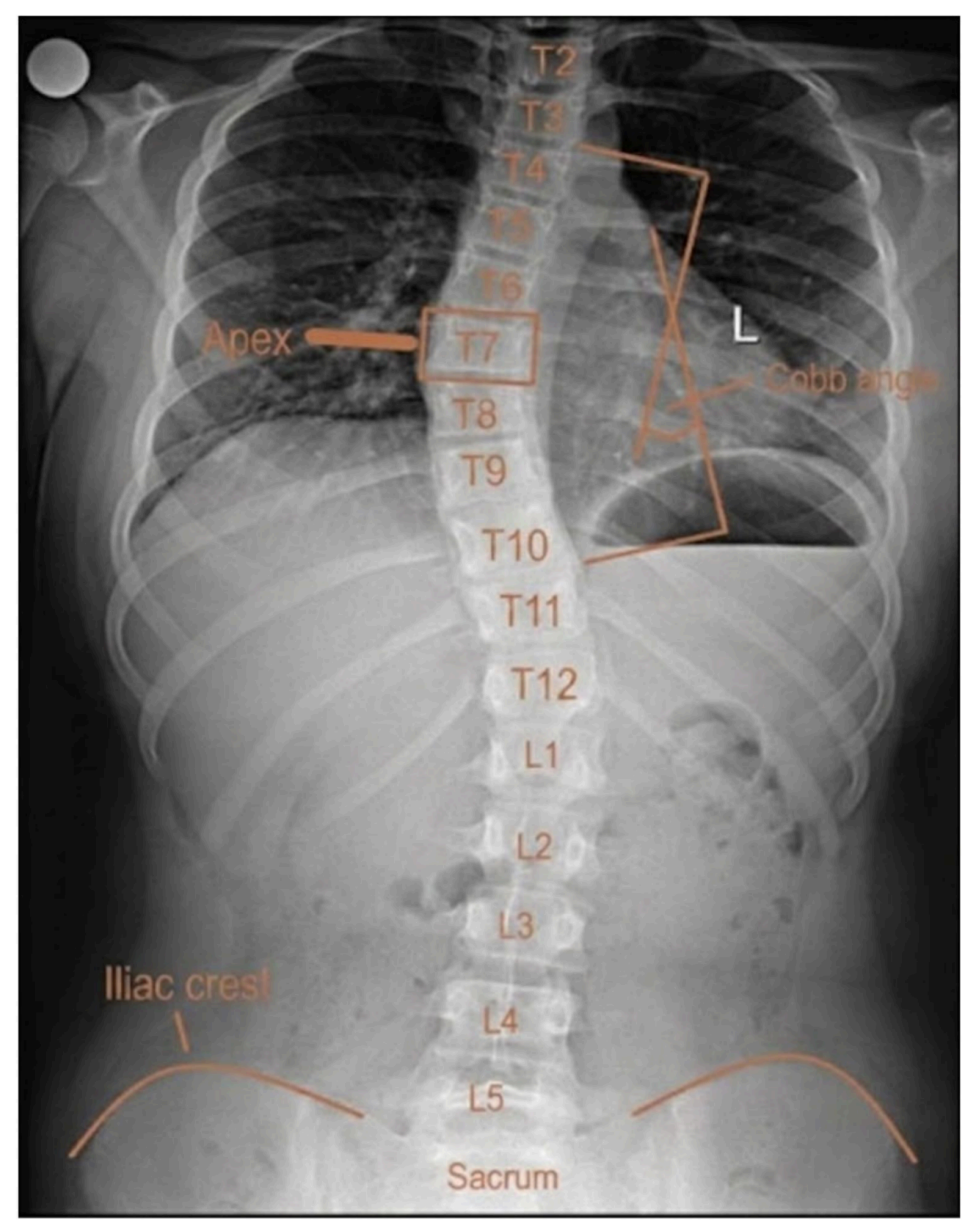
Representative standing anteroposterior (AP) thoracolumbar radiograph demonstrating digital Cobb angle measurement and anatomical landmarks. The structural thoracic curve exhibits an apex at T7 (orange box), with the upper end vertebra (T4, superior endplate) and lower end vertebra (T10, inferior endplate) defining the Cobb angle (measured at 28°, indicating moderate scoliosis). Anatomical reference points including the thoracic spine (T2–T12), lumbar spine (L1–L5), sacrum, and bilateral iliac crests (demarcated by orange lines for Risser staging assessment) are labeled. L= left.

Standardized standing anteroposterior (AP) full-spine radiographs were obtained using a fixed source-to-image distance (SID) of 150–200 cm to minimize magnification distortion. Patients were positioned in weight-bearing, barefoot, with knees fully extended and hips level. Arm position was placed relaxed down by the sides of the body, or slightly away from the torso. All DICOM images were processed and measured using RadiAnt DICOM Viewer 2025.2 (Medixant, Poznań, Poland). Cobb angles were calculated digitally by identifying the superior endplate of the most tilted upper end vertebra (UEV) and the inferior endplate of the most tilted lower end vertebra (LEV) defining the structural curve. Intersecting lines perpendicular to these endplates were automatically drawn by the software to determine the Cobb angle in degrees (°). The apical vertebra was identified according to strict Scoliosis Research Society (SRS) guidelines as the segment located at the maximum lateral deviation from the center sacral vertical line (CSVL). Axial vertebral rotation was evaluated independently using the Nash–Moe grading system and was not used as the defining criterion for apical position. In patients presenting structural double or multiple curves, the major curve was defined as the structural curve with the largest Cobb angle magnitude, which was subsequently used for localized categorization and severity stratification (Figure 1).

Because dynamic flexibility radiographs (e.g., side-bending views) were not performed as part of this screening protocol, structural and non-structural curves were not formally differentiated. All identified coronal curves with a Cobb angle ≥ 10° on standard standing AP radiographs were included in the analysis. Curve direction (right vs. left) was assigned strictly according to the convexity side of this primary major curve.

Skeletal maturity was concurrently graded using the classic Risser staging system (stages 0–5) based on the degree of apophyseal ossification across the iliac crest from anterior-superior to posterior-superior iliac spine [30,34].

To assess the internal consistency and reproducibility of the radiological measurements, all radiographs across the entire cohort (n = 233) were re-evaluated by the same blinded observer after a 2-month interval, experienced radiologist with 15 years of experience in conventional radiology. Intra-observer agreement was quantified using a two-way mixed-effects, single-rating, absolute-agreement Intraclass Correlation Coefficient (ICC); inter-observer reliability was not evaluated. Intra-observer reliability for digital Cobb angle measurements demonstrated excellent consistency, with an intraclass correlation coefficient (ICC) of 0.94 (95% CI: 0.92–0.97). The assessment of skeletal maturity using the Risser staging system also yielded high intra-observer agreement with a linearly weighted Cohen’s kappa of к_w_ = 0.946 (95% CI: 0.936–0.974) across the evaluated cohort, confirming high reproducibility of ordinal skeletal staging.

### 2.3. Statistical Analysis

Statistical analysis was conducted using GraphPad Prism version 10.3.1 (GraphPad Software, Boston, MA, USA) and SPSS version 28.0 (IBM Corp., Armonk, NY, USA).

Univariate associations were evaluated using the Pearson chi-square test or Fisher’s exact test for categorical variables. To account for potential confounding variables (such as age and sex) and identify independently associated factors of scoliosis severity, multivariable ordinal logistic regression analysis was performed, incorporating Cobb angle severity categories as the dependent variable. *p*-values < 0.05 were considered statistically significant.

Continuous variables are presented as mean ± standard deviation, and categorical variables as frequencies and percentages. To evaluate variables associated with the binary presence of scoliosis (Cobb angle ≥ 10°), a multivariable binary logistic regression model was fitted. An ordinal logistic regression model was constructed for entire cohort (n = 233) to analyze factors associated with disease severity categorized across four ordered categories: normal (non-scoliosis < 10°), mild (10–24°), moderate (25–39°), and severe (≥40°). Adjusted odds ratios (aOR) with corresponding 95% confidence intervals (CI) were derived. To ensure the validity and stability of the multivariable ordinal logistic regression model, full model diagnostics were evaluated. The proportional odds assumption was formally evaluated using the Brant test. Multicollinearity among covariates (particularly between age and Risser stage) was assessed using Variance Inflation Factors (VIF), and overall model goodness-of-fit was evaluated using Pseudo-R^2^ metrics (Table A2).

The primary endpoint was scoliosis severity, defined according to Cobb angle measurements and classified across four ordered levels.

Secondary endpoints included:

1. Curve localization was determined based on the affected anatomical segment of the vertebral column: thoracic, thoracolumbar, lumbar.
  In the presence of multiple curves, we determined the one at which the main structural abnormality was present (major curve).
2. Curve direction: right-sided convexity, curvature towards the right, left-sided convexity, curvature towards the left.
3. Skeletal maturity: was determined based on the level of ossification and fusion of the iliac crest apophyses in 5 stages: Risser stage (0–5).
4. The presence and localization of characteristic radiographic signs of spina bifida occulta were determined based on the affected vertebra: L5, S1, combined L5 + S1 involvement.

## 3. Results

Of the 233 examined individuals, 88 (37.8%) had Cobb angles below 10°, while 145 (62.2%) were diagnosed with scoliosis (Cobb angle ≥ 10°). Among the scoliotic patients, the severity distribution was as follows: 96 patients (66.2%) presented with mild scoliosis (≥10–24°), 43 (29.7%) with moderate scoliosis (25–39°), and 6 (4.1%) with severe scoliosis (≥40°) (Figure A1).

Regarding overall curve localization, among the 145 scoliotic patients, thoracic localization was identified in 48.3% (70/145), thoracolumbar junction involvement in 45.5% (66/145), and lumbar localization in 6.2% (9/145). A statistically significant association was observed between curve localization and scoliosis severity (*p* = 0.007). Thoracolumbar curves were 56.3% predominantly mild (54/96), whereas thoracic localization predominated in moderate 67.4% (29/43) and 66.7% (4/6) severe scoliosis cases. Detailed distributions by severity group are summarized in Table 1.

Right-sided curvature was observed in 85 patients (58.6%) comprising 51 mild (60%), 30 (35.2%) moderate, and 4 (4.7%) severe cases. Left-sided curvature was observed in 60 patients (41.4%), of whom 75% had mild, 21.7% moderate, and 3.3% severe scoliosis. No statistically significant association was found between curve direction and scoliosis severity (*p* = 0.16).

Within the scoliosis group, a 3.14:1 female-to-male ratio was observed (110 females, 35 males). Sex was not significantly associated with scoliosis severity (*p* = 0.16). Among male patients, 77.1% (27/35) had mild, 17.1% (6/35) moderate, and 5.7% (2/35) severe scoliosis. Among female patients, 62.7% (69/110) had mild, 33.6% (37/110) moderate, and 3.6% (4/110) severe scoliosis (Table 1). When comparing scoliotic and non-scoliotic individuals, the female proportion was significantly higher in the scoliosis group (110/145, 75.9%) compared to the non-scoliotic group (42/88, 47.7%; *p* < 0.0001, Table 2).

Spina bifida occulta (SBO) was identified in 17 of the 145 scoliotic patients (11.7%) and in 13 of the 88 non-scoliotic individuals (14.8%). In all cases, the defect was located at the L5 or S1 vertebral level, in two patients both levels were involved.

As is evident from Table 1, the presence of spina bifida was not significantly associated with scoliosis severity (*p* = 0.192), although mild and moderate curves were relatively more frequent in affected individuals. The spina bifida occulta anatomical localization was not significantly associated with scoliosis curve localization (*p* = 0.11). In scoliotic patients, SBO was located at S1 in 82.4% (14/17), at L5 in 5.9% (1/17), and at both L5 + S1 levels in 11.8% (2/17). In non-scoliotic patients, SBO was present at S1 in 61.5% (8/13) and at L5 in 38.5% (5/13), with no combined L5 + S1 cases (Table 2).

The overall average age was 13.7± 2.8 years (13.1 ± 2.9 years in non-scoliotic individuals vs. 14.1 ±2.7 years in scoliotic patients). Age was not significantly associated with scoliosis presence (*p* = 0.14) or curve severity (*p* = 0.7, Table 1 and Table 2).

Skeletal maturity (Risser stage) overall followed expected age-related ossification patterns in both sexes (*p* < 0.0001, Table 3 and Table 4). However, we did not identify significant differences in Risser stage distribution between scoliotic and non-scoliotic adolescents (males: *p* = 0.33; females: *p* = 0.05). Especially, a subset of young adult females (>17 years) with severe scoliosis presented delayed skeletal maturation (Risser stage 2 in 2 patients), whereas all non-scoliotic young adult females achieved complete skeletal maturity (Risser stage 5; Table 4 and Table 5).

The radiological characteristics of the Risser sign were analyzed in relation to the presence of scoliosis. In accordance with literature, the ossification scale was adjusted to age- and sex-specific reference values [30,35,36,37].

In male patients with scoliosis, all preadolescent boys presented a Risser stage 0 (100%). Among adolescent boys, Risser stages ranged from 0 to 4, with Risser stage 4 being the most frequent (6/23). In young adult males with scoliosis, the Risser stage was 5 in all cases (*p* < 0.0001).

In non-scoliotic male patients, a total of 46 spinal radiographs were analyzed. In preadolescent boys, the Risser stage was 0 in all cases. Among adolescent boys, Risser stages varied between 0 and 4, most frequently stages 2 and 3 (16/27). In young adult males, the Risser stage was 5 in all cases (*p* < 0.0001) (Table 3).

The comparison of Risser stages between scoliotic and non-scoliotic male patients revealed that in preadolescent boys, the Risser stage was 0 on all spinal radiographs. In both groups, ossification had not yet started before adolescence, which is considered physiologically normal. A statistically significant difference was not observed between scoliotic and non-scoliotic boys with respect to Risser stages (*p* = 0.33), as evident from Table 3.

In female patients with scoliosis, all preadolescent girls presented a Risser stage 0. Among adolescent girls, Risser stages ranged from 0 to 4, with Risser stage 3 being the most frequent (25/83). In young adult females with scoliosis, Risser stages 2, 4, and 5 were observed, with stage 5 occurring most frequently (13/21).

In the group of non-scoliotic female patients, 42 spinal radiographs were analyzed. The observed Risser stages were equivalent to expected ossification stages for the respective age groups.

Among both scoliotic and non-scoliotic preadolescent girls, all spinal radiographs demonstrated a Risser stage 0. Ossification had not commenced in either group prior to the age of 9 years.

In adolescent girls, in the scoliotic group, 24% (20/83) had a Risser stage 0, compared with 43.7% (14/32) in the non-scoliotic group. A Risser stage 4 was observed in 4.8% (4/83) of scoliotic patients, whereas this value occurred in 12.5% (4/32) of non-scoliotic patients (Table 4).

Differences were also observed among young adult females. In 38% (8/21) of scoliotic cases the Risser stage was lower than 5, and in 9% (2/21) of cases a Risser stage 2 was identified.

In contrast, all non-scoliotic young adult female patients presented a Risser stage 5 (Table 4).

A statistically significant difference was not observed between scoliotic and non-scoliotic girls, with respect to Risser stages (*p* = 0.05), as evident from Table 4.

The association between scoliosis severity and Risser stage was analyzed with respect to sex.

In cases of mild scoliosis, all preadolescent boys exhibited a Risser stage 0. Among adolescent boys, Risser stages ranged from 0 to 4, with Risser stages 1 and 2 observed in 50% of cases. In moderate scoliosis, all preadolescent boys also presented with a Risser value of 0. In adolescent males, Risser stages ranged from 0 to 4, with Risser stage 4 occurring in approximately 60% of cases. Among adolescent males with severe scoliosis, two patients were identified, with Risser values of 2 and 4, respectively (Table 5).

In female patients, the Risser stage was 0 in all individuals prior to adolescence. During adolescence, the distribution of Risser stages was consistent with physiological expectations.

In cases of severe scoliosis, all young adult female patients presented a Risser stage 2 (Table 4 and Table 5).

To establish independently associated factors of scoliosis severity and control for potential confounders, a multivariable ordinal logistic regression model was performed. Curve localization, sex, age, and Risser stage were included as independent variables (Table A2).

To evaluate independently associated factors for curve severity and address potential univariable confounding, multivariable linear regression analysis was performed for total and region-specific Cobb angles. Within the scoliotic cohort (n = 145), age (β = 1.99, 95% CI: 0.86 to 3.12, *p* < 0.001) and Risser stage (β = −2.67, 95% CI: −4.66 to −0.67, *p* = 0.009) were identified as multivariable associated factors of maximum Cobb angle magnitude. Patient sex (*p* = 0.134) and the presence of spina bifida occulta (*p* = 0.721) showed no significant independent association with curve severity (Table A1).

Multivariable ordinal logistic regression revealed that thoracic curve localization (aOR = 4.64, 95% CI: 2.56–8.13, *p* < 0.001) and female sex (aOR = 3.06, 95% CI: 1.73–5.41, *p* < 0.001) were independently associated with higher scoliosis severity categories. Age per year increase demonstrated a borderline independent association with severity (aOR = 1.17, 95% CI: 1.00–1.37, *p* = 0.050). Neither Risser stage (aOR = 0.89, 95% CI: 0.67–1.19, *p* = 0.434) nor the presence of spina bifida occulta (aOR = 1.02, 95% CI: 0.48–2.14, *p* = 0.965) demonstrated statistically significant independent effects after multivariable adjustment (Table A2).

Subgroup analysis based on anatomical region demonstrated distinct patterns of association with thoracic curve, age was positively associated with curve magnitude (β = 2.12, 95% CI: 0.45 to 3.78, *p* = 0.013), whereas higher Risser stage was inversely associated with Cobb angle (β = −3.11, 95% CI: −6.10 to −0.13, *p* = 0.041).

For thoracolumbar curves, similar directional trends were observed for age (β = 1.14, *p* = 0.134) and Risser stage (β = −1.21, *p* = 0.373), though neither reached statistical significance (Table A3).

Model diagnostics confirmed the appropriateness and stability of the ordinal logistic regression framework (Table A2). The Brant test supported the proportional odds assumption (χ^2^ = 8.12, *p* = 0.150), indicating that the effect of covariates remained consistent across scoliosis severity thresholds. Furthermore, multicollinearity was ruled out, with all Variance Inflation Factor (VIF) values remaining well below threshold limits (VIF < 3.8, e.g., age and Risser stage exhibited acceptable collinearity), and overall model goodness-of-fit was confirmed. However, findings within smaller subgroups—specifically severe scoliosis (n = 6) and primary lumbar curves (n = 9)—remain exploratory and subject to sparse-data constraints.

## 4. Discussion

In this retrospective observational study, we characterized the baseline epidemiological and radiographic profile of a pediatric, adolescent, and young adult referral cohort. Our findings demonstrate a significant association between chronological age and current curve severity, as reflected by higher Cobb angles at the presentation in older patients. In alignment with our cross-sectional design, these observations represent the baseline correlates of deformity severity rather than the predictive markers of prospective curve progression.

In the present study, 62% of participants demonstrated Cobb angles greater than 10°, indicating the radiographic evidence of scoliosis within this referral-based radiographic cohort. This proportion does not represent population-level prevalence, but it rather reflects the distribution of scoliosis among pediatric, adolescent and young adult patients referred for spinal imaging due to the clinical suspicion of scoliosis. Accordingly, the findings should be interpreted in the context of referral and selection bias inherent to hospital-based radiological datasets.

The severity distribution showed that 66% of scoliotic cases were classified as mild (10–24°), 29.7% as moderate (25–39°), and 4.1% as severe (≥40°). While the predominance of mild scoliosis is consistent with screening-based literature, the relatively higher proportion of moderate cases likely reflects differences in referral thresholds, diagnostic timing, and case selection.

The mean Cobb angle (21.82°) further supports the predominance of mild scoliotic curvature in this cohort. This value is lower than those reported in tertiary care and surgical populations, such as Yilmaz et al. (28.11° ± 9.35°) and Doi et al. (32.9° ± 11.9°), reinforcing the impact of cohort composition on radiographic curve severity profiles. These discrepancies highlight the need for caution when comparing referral-based cohorts with surgical or intervention-oriented populations, as they represent different stages of disease evolution [13,38].

A statistically significant association was identified between scoliosis localization and severity (*p* = 0.007). Thoracic curves were more frequently associated with higher Cobb angles, whereas thoracolumbar curves were predominantly mild. This may reflect biomechanical differences, including the stabilizing effect of the rib cage in thoracic deformities and the increased flexibility of the thoracolumbar junction, potentially influencing curve behavior during growth [4,39].

Thoracic and thoracolumbar curves represented the dominant patterns, while lumbar scoliosis was relatively rare, consistent with established classification-based descriptions of adolescent idiopathic scoliosis patterns [10].

In addition, multivariable ordinal logistic regression across the entire referral cohort indicated that primary thoracic curve location (aOR = 4.64, 95% CI: 2.56–8.13, *p* < 0.001) and the female sex (aOR = 3.06, 95% CI: 1.73–5.41, *p* < 0.001) were the principal independently associated factor with higher scoliosis severity categories (Table A2). No significant association was observed between curve direction and severity (*p* = 0.16), although right-sided convexity predominated. This aligns with established epidemiological patterns describing right thoracic curves as the most frequent presentation in adolescent idiopathic scoliosis, with minor inter-population variability reported in the literature.

The female-to-male ratio was 3.14:1 among scoliotic patients, consistent with well-documented female predominance in adolescent scoliosis. Sex was not significantly associated with curve severity, moderate and severe curves were more frequently observed in females, potentially reflecting differences in growth velocity, pubertal timing, and skeletal maturation [3,6,40].

Radiological signs consistent with spina bifida were identified in 11.7% of scoliotic patients, predominantly at the L5 and S1 levels. Although no significant association was observed between spina bifida presence and scoliosis severity (*p* = 0.19), a higher proportion of moderate curves was noted in affected individuals, suggesting a possible biomechanical contribution of posterior arch defects to segmental stability. However, causal inference is limited due to the retrospective design.

A significant association was not observed between spina bifida localization and scoliosis localization (*p* = 0.11). Although, S1 involvement was more frequently observed in thoracic and thoracolumbar curves, whereas L5 involvement was present in lumbar scoliosis. Combined L5–S1 defects were observed exclusively in scoliotic patients. These findings differ from previous reports describing predominantly lumbar involvement, suggesting possible population-specific anatomical variability or differences in imaging selection criteria. However, given the cross-sectional nature of this study and the relatively small subgroup sizes, these findings must be interpreted strictly as exploratory rather than indicative of a direct causal or mechanical relationship.

The average age of scoliotic patients (14.1 years) corresponded with the known peak incidence of adolescent idiopathic scoliosis during the pubertal growth spurt. The highest frequency of cases at approximately 15 years further supports this developmental window as a critical period for clinical detection. The absence of a statistically significant age trend between scoliotic and non-scoliotic patients (*p* = 0.1) likely reflects the referral-based structure of the cohort.

Risser stage distribution was consistent with expected skeletal maturation patterns, with stage 0 predominating in pediatric patients and stage 5 in young adults. Among adolescents, variability (0–4) reflected normal pubertal ossification progression. Overall, skeletal maturity followed physiological expectations in both scoliotic and non-scoliotic groups.

Interestingly, a very small subset of young adult female patients with severe scoliosis demonstrated lower-than-expected Risser stages (e.g., stage 2), suggesting delayed skeletal maturation relative to chronological age. The extremely small sample size (n = 2) and cross-sectional design preclude any generalization, and this finding should be treated strictly as preliminary and exploratory, warranting further evaluation in prospective longitudinal cohorts.

To our knowledge, this study provides a comprehensive radiographic characterization of scoliosis in a referral-based pediatric, adolescent and young adult cohort, integrating curve severity, localization, skeletal maturity (Risser stage), and the presence of spina bifida for a more comprehensive radiological evaluation.

Curve localization, sex, age, and Risser stage were included as independent variables (Table A2) [21,25]. Thoracic curve localization remained an independently associated factor of higher scoliosis severity compared to thoracolumbar localization, corroborating recent multivariable associational models [24,26].

### 4.1. Clinical Relevance

These findings emphasize the utility of incorporating region-specific radiographic curve analysis alongside skeletal maturity assessment in the initial evaluation of pediatric, adolescent and young adult scoliosis. Within the thoracic curve subgroup, lower Risser stages were cross-sectionally associated with larger Cobb angles (β = −3.11°/stage), highlighting the regional relationship between structural curve magnitude and ossification stage at clinical presentation [11,14,39].

The results further support the role of structured radiographic evaluation in providing comprehensive cross-sectional characterization of spinal curvature at initial clinical evaluation, particularly across different phases of skeletal growth [4,11,41].

### 4.2. Limitations

This study has several limitations that warrant consideration. The non-idiopathic etiologies of scoliosis (such as congenital vertebral malformations or neuromuscular conditions) were excluded during initial clinical screening; our findings apply strictly to idiopathic scoliosis and cannot be generalized to secondary or syndromic spinal deformities. The comparison group (<10°) consists of individuals referred for suspected spinal asymmetry or postural concern rather than healthy unselected population controls, reflecting an outpatient referral baseline. Furthermore, because dynamic flexibility radiographs (such as side-bending views) were not routinely obtained, all scoliotic curves were evaluated using standing AP radiographs alone without formally differentiating structural from non-structural components.

Its retrospective and single-center design introduces inherent selection and referral biases, limiting generalizability to the broader population. Additionally, the cross-sectional nature of the analysis precludes assessment of longitudinal curve progression and causal inference. Future studies incorporating prospective longitudinal data are required to validate these cross-sectional findings and evaluate their clinical utility over time. Although intra-observer reliability was excellent, inter-observer reliability was not evaluated. Despite adjusting for key covariates (age, sex, Risser stage, spina bifida) in multivariable linear and ordinal regression models, residual confounding factors (such as exact menarchal status or truncal rotation) may still influence observed associations.

Finally, the relatively small number of severe scoliosis cases and isolated lumbar curves limited statistical power for specific subgroup analyses and may reduce the reliability of observations related to advanced scoliotic curve patterns.

## 5. Conclusions

Scoliosis represents a frequent indication for radiological evaluation in pediatric, adolescent and young adult populations, with this study reflecting a referral-based radiographic cohort rather than true population prevalence.

Thoracic and thoracolumbar curves were the predominant patterns, with thoracic localization showing an association greater with curve magnitude.

A marked female predominance was confirmed among scoliotic patients, consistent with established epidemiological patterns in adolescent idiopathic scoliosis.

Multivariable ordinal logistic regression indicated that primary thoracic curve location and the female sex were the principal independently associated factor with higher scoliosis severity categories. Subgroup analysis based on anatomical region demonstrated distinct patterns of association for thoracic curve, age was positively associated with curve magnitude, whereas higher Risser stage was inversely associated with Cobb angle. Skeletal maturity assessment using the Risser classification demonstrated overall age-appropriate ossification patterns.

## Figures and Tables

**Table 1:** Demographic and Clinical Characteristics According to Scoliosis Severity.

	SCOLIOSIS			
	10–24°	25–39°	≥40°	*p* (<0.05)
**LOCATION**				
THORACIC	37	29	4	*p* = 0.007
LUMBAR	5	4	0	
THORACOLUMBAR	54	10	2	
**CURVE DIRECTION**				
RIGHT	51	30	4	*p* = 0.160
LEFT	45	13	2	
**GENDER**				
MALE	27	6	2	*p* = 0.160
FEMALE	69	37	4	
**SPINA BIFIDA**				
(+)	9	8	0	*p* = 0.192
(−)	87	35	6	
**AGE (YEARS)**				
0–9	7	0	0	*p* = 0.7
10–12	23	10	1	
13	13	6	1	
14	12	5	1	
15	17	8	0	
16	10	5	0	
17–18	7	6	2	
>19	7	3	1	

**Table 2:** Patient Characteristics in the Scoliosis and Non-Scoliosis Groups.

	SCOLIOSIS	NON-SCOLIOSIS	*p* (<0.05)
**GENDER**			
MALE	35	46	*p* < 0.0001
FEMALE	110	42	
**SPINA BIFIDA**			
(+)	17	13	*p* = 0.7
(−)	128	75	
**SPINA BIFIDA LOCATION**			
L5	1	5	*p* = 0.05
S1	14	8	
L5 + S1	2	0	
**AGE (YEARS)**			
0–9	7	13	*p* = 0.140
10–12	34	24	
13	20	10	
14	18	11	
15	25	10	
16	15	11	
17–18	15	4	
>19	11	5	

**Table 3:** Distribution of Male Patients by Risser Stage, Age Group, and Scoliosis Status.

MALE	RISSER	0	1	2	3	4	5
SCOLIOSIS	AGE (YEARS)						
	0–12	10	0	0	0	0	0
	13–18	2	5	5	5	6	0
	>19	0	0	0	0	0	2
**NON-SCOLIOSIS**							
	0–12	17	0	0	0	0	0
	13–18	6	2	8	8	3	0
	>19	0	0	0	0	0	2

**Table 4:** Female Risser Stages According to Age and Scoliosis Status.

FEMALE	RISSER	0	1	2	3	4	5
SCOLIOSIS	AGE (YEARS)						
	0–9	6	0	0	0	0	0
	10–16	20	22	12	25	4	0
	>17	0	0	2	0	6	13
**NON-SCOLIOSIS**							
	0–9	5	0	0	0	0	0
	10–16	14	3	7	4	4	0
	>17	0	0	0	0	0	5

**Table 5:** Relationship Between Scoliosis Severity and Risser Stage by Sex.

	MALE RISSER				FEMALE RISSER			
SCOLIOSIS	AGE (YEARS)	0	1–3	4–5	AGE (YEARS)	0	1–3	4–5
10–24°	0–12	9	0	0	0–9	6	0	0
	13–18	1	13	2	10–16	12	36	3
	>19	0	0	2	>17	0	0	12
25–39°	0–12	1	0	0	0–9	0	0	0
	13–18	1	1	3	10–16	8	21	1
	>19	0	0	0	>17	0	0	7
≥40°	0–12	0	0	0	0–9	0	0	0
	13–18	0	1	1	10–16	0	2	0
	>19	0	0	0	>17	0	2	0

## Data Availability

The data presented in this study are unavailable due to privacy and ethical restrictions.

## Appendix A

**Table A1:** Multivariable linear regression analysis of maximum Cobb angle values.

Predictor Variable	β	95% CI	*p*-Value
Sex (Female)	3.09	−0.96 to 7.14	0.134
Age (years)	1.99	0.86 to 3.12	<0.001
Risser stage	−2.67	−4.66 to −0.67	0.009
Spina Bifida (present)	−0.96	−6.29 to 4.36	0.721

**Table A2:** Multivariable ordinal logistic regression analysis of overall cohort scoliosis severity across four ordered categories (n = 233).

Predictor Variable	β Coefficient	aOR	95% CI	*p*-Value	Multicollinearity (VIF)
Thoracic Location (vs. Thoracolumbar/Lumbar)	1.536	4.64	2.56 to 8.13	<0.001	1.05
Age (per year increase)	0.158	1.17	1.00 to 1.37	0.05	3.72
Risser stage (per stage increase)	−0.116	0.89	0.67 to 1.19	0.434	3.75
Sex (Female vs. Male)	1.118	3.06	1.73 to 5.41	<0.001	1.05
Spina Bifida Occulta (Present)	0.017	1.02	0.48 to 2.14	0.965	1.01

Model Overall Fit: Likelihood-Ratio χ^2^ (5) = 61.91, *p* < 0.001 (Model Log-Likelihood: −234.47 vs. Null Log-Likelihood: −265.43). Goodness-of-Fit/Pseudo R^2^: Nagelkerke R^2^ = 0.260 (26.0%), Cox & Snell R^2^ = 0.233 (23.3%), McFadden R^2^ = 0.117 (11.7%). Proportional-Odds Assumption (Brant Test): χ^2^ = 8.12, *p* = 0.150. Cut-off Threshold Parameters: α1 (Normal/Mild) = 1.152, α2 (Mild/Moderate) = 3.245, α3 (Moderate/Severe) = 5.812. Severe Cases (n = 6, Cobb ≥ 40°): Female: 66.7%, SBO present: 0.0%, Risser 2–3: 66.7%, Thoracic: 66.7%.

**Table A3:** Multivariable regression analysis by specific spine region Cobb angles.

Variable	Thoracic Cobb β (N = 70)	95% CI	*p*-Value	Thoracolumbar Cobb β (N = 66)	95% CI	*p*-Value
Sex (Female)	−0.22	−7.41 to 6.97	0.952	2.38	−2.13 to 6.92	0.298
Age (years)	2.12	0.45 to 3.78	0.013	1.14	−0.36 to 2.651	0.134
Risser stage	−3.11	−6.10 to −0.13	0.041	−1.21	−3.89 to 1.48	0.373
Spina Bifida	3.29	−7.28 to 13.86	0.537	−0.97	−6.78 to 4.85	0.741
